# Phyllodiaptomus (Phyllodiaptomus) roietensis, a new diaptomid copepod (Copepoda, Calanoida) from temporary waters in Thailand and Cambodia, with a key to the species

**DOI:** 10.3897/zookeys.911.38496

**Published:** 2020-02-12

**Authors:** La-orsri Sanoamuang, Santi Watiroyram

**Affiliations:** 1 Applied Taxonomic Research Center, Faculty of Science, Khon Kaen University, Khon Kaen 40002, Thailand; 2 International College, Khon Kaen University, Khon Kaen 40002, Thailand; 3 Division of Biology, Faculty of Science, Nakhon Phanom University, Nakhon Phanom 48000, Thailand

**Keywords:** Diaptomidae, freshwater, rare species, Southeast Asia, taxonomy, temporary water bodies

## Abstract

Phyllodiaptomus (Phyllodiaptomus) roietensis**sp. nov.** was collected from temporary water bodies in Roi Et and Nakhon Ratchasima provinces in northeastern Thailand and Kampong Thom Province in central Cambodia. The new species is closely related to Phyllodiaptomus (P.) surinensis Sanoamuang & Yindee, 2001 in that it shares common morphological characters in the males: urosomites 2–3, P5 intercoxal sclerite, right P5 Exp-2, and left P5 Exp. Minor differences on the right antennule, right caudal ramus, P5 basis and Enp exist. The females differ in their Pdg 5, genital double-somite, and P5. An updated key to the species of the genus *Phyllodiaptomus* Kiefer, 1936 is provided.

## Introduction

The genus *Phyllodiaptomus* Kiefer, 1936, is among the most common freshwater copepods in Southeast Asia (Sanoamuang 1999). To date, eleven valid species have been recorded in Asia (Walter and Boxshall 2018): Phyllodiaptomus (Phyllodiaptomus) blanci (Guerne & Richard, 1896) from Uzbekistan; P. (Ctenodiaptomus) annae (Apstein, 1907) from Sri Lanka; P. (P.) tunguidus Shen & Tai, 1964 from China; P. (P.) longipes Kiefer, 1965 from Indonesia; P. (C.) sasikumari Ranga Reddy & Venkateswarlu, 1989 and P. (C.) wellekensae Dumont & Ranga Reddy, 1993 from India; P. (C.) praedictus Dumont & Ranga Reddy, 1994, P. (P.) christineae Dumont, Ranga Reddy & Sanoamuang, 1996, P. (P.) surinensis Sanoamuang & Yindee, 2001, and P. (P.) thailandicus Sanoamuang & Teeramaethee, 2006 from Thailand; and P. (P.) irakiensis Khalaf, 2008 from Iraq. In addition, Alekseev et al. (2013) reported P. (C.) praedictus sulawesensis as a subspecies of *P.* (*C*) *praedictus* from Indonesia; this subspecies was later found in the Philippines (Guinto et al. 2018).

**Figure 1.**
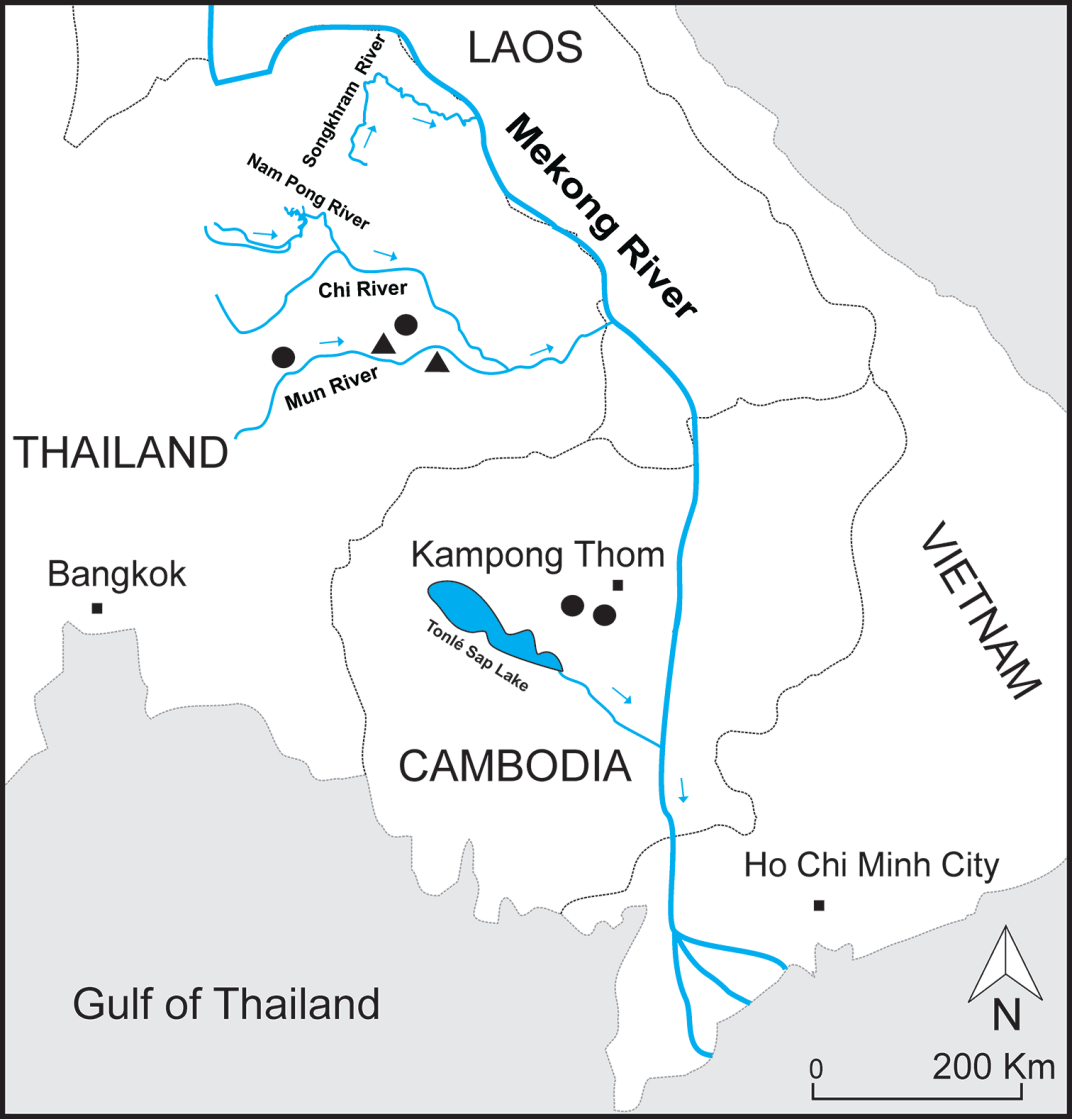
Distribution of Phyllodiaptomus (P.) roietensis sp. nov. and P. (P.) surinensis. Key: black square = city, black circle = P. (P.) roietensis sp. nov., black triangular = P. (P.) surinensis, blue arrows indicate water flow direction.

During seasonal sampling collections of freshwater copepods from several localities in Thailand and Cambodia, we encountered another hitherto unknown species of *Phyllodiaptomus*. In this paper, we describe Phyllodiaptomus (P.) roietensis sp. nov. from two localities in Roi Et and Nakhon Ratchasima provinces, northeast Thailand, and two localities in Kampong Thom Province in central Cambodia (Fig. 1).

## Materials and methods

Samples were collected using a plankton net with a mesh size of 60 µm and preserved immediately in 70% ethanol. Adult copepods were selected under an Olympus SZ51 stereomicroscope at 40-x magnification and placed in a mixture of glycerol and 70% ethanol (ratio ~ 1:10 v/v). After 10 min the animals were transferred to pure glycerol. The animals were dissected and prepared in a glycerin-mounted slide under a stereomicroscope at 40–100-x magnifications. The dissected specimens were mounted in pure glycerin on a glass slide and sealed under a cover glass with transparent nail varnish. All un-dissected specimens were stored in 70% ethanol in 1.5 mL microtubes.

All appendages and body ornamentation were examined at 1000-x magnification under an Olympus CX31 compound microscope. The drawings were made using an Olympus U-DA drawing tube mounted on a compound microscope. The final versions of the drawings were made using the CORELDRAW 12.0 graphic program.

Specimens for scanning electron microscopy (SEM) were dehydrated in an ethanol series (50%, 70%, 80%, 90%, 95%, 100%, and 100%) for 15 min at each concentration. Specimens were dried in a critical-point dryer and were mounted on stubs using adhesive tape under a stereomicroscope. Dried specimens were coated with gold in a sputter-coater. The SEM photographs were taken using a scanning electron microscope (FEI Helios NanoLab G3 CX).

Specimens were deposited at the Natural History Museum, London, United Kingdom (**NHMUK**) and at the Applied Taxonomic Research Center, Khon Kaen University (Thailand) (**KKU**).

Abbreviations used in this paper are as follows:

**ae** aesthetasc;

**Enp** endopod;

**Exp** exopod;

**Exp/Enp-n** exopodal segment n/endopodal segment n;

**Pdg** pediger;

**Pdg 1**–**5** pedigers 1–5;

**P1**–**P5** legs 1–5;

**sp** spine.

The descriptive terminology follows Huys and Boxshall (1991).

## Taxonomic section

### Order Calanoida Sars, 1903

#### Family Diaptomidae Baird, 1850

**Sub-family Diaptominae Kiefer, 1932**

**Genus *Phyllodiaptomus* Kiefer, 1936**

**Subgenus Phyllodiaptomus Dumont, Ranga Reddy & Sanoamuang, 1996**

##### Phyllodiaptomus (P.) roietensis sp. nov.

Taxon classificationAnimaliaCalanoidaDiaptomidae

0B9F97DA-0E9E-5345-88D4-29E2AF212FBE

http://zoobank.org/59131C6D-A0DE-4BE0-9383-20C0DA8A709D

**Figure 2.**
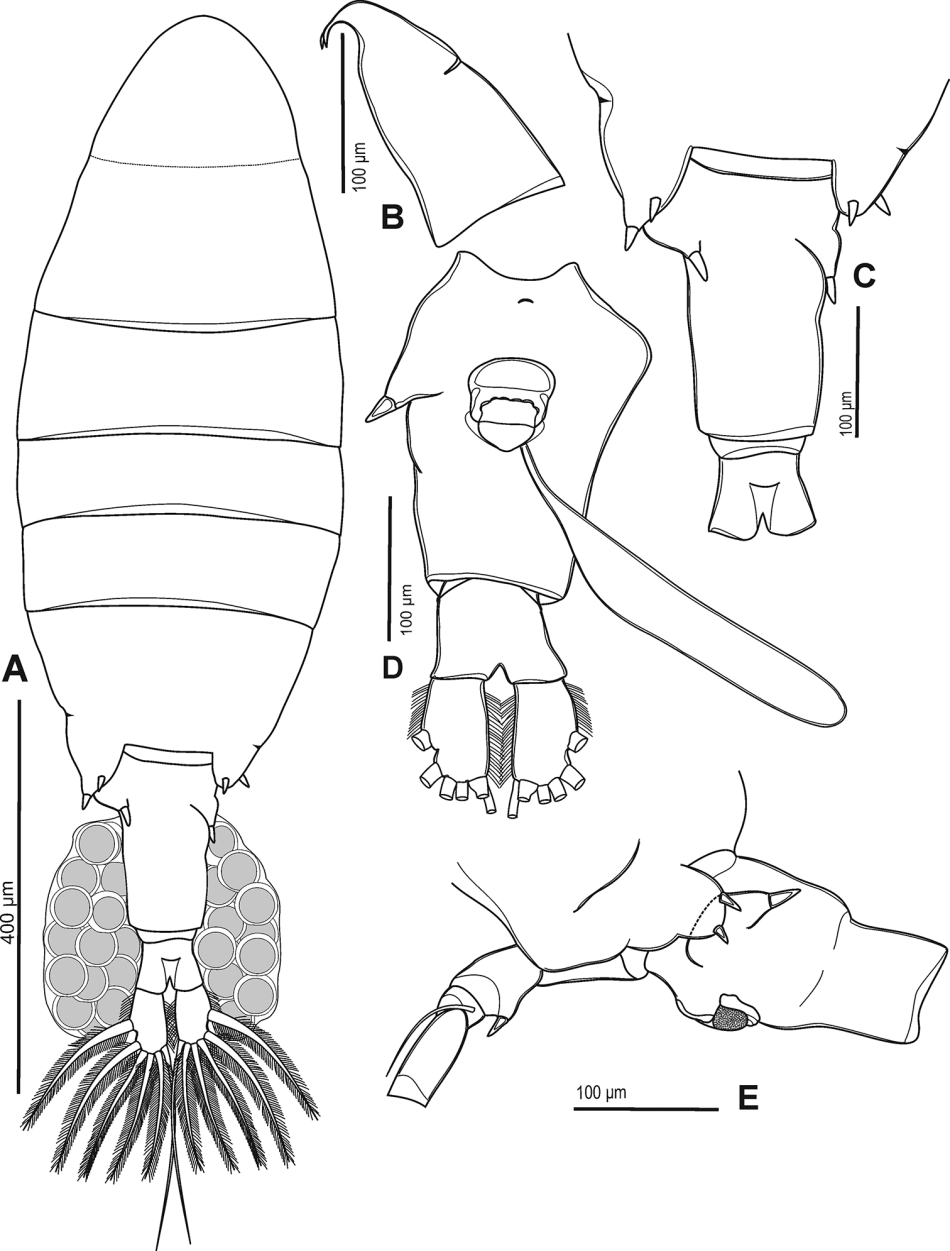
Phyllodiaptomus (P.) roietensis sp. nov., female: **A** habitus, dorsal view **B** cephalosome with rostrum, lateral view **C** lateral wings on Pdg 5 and urosome (without caudal rami), dorsal view **D** urosome, ventral view **E**Pdg 5 with P5 and genital double-somite, lateral view from left side.

**Figure 3.**
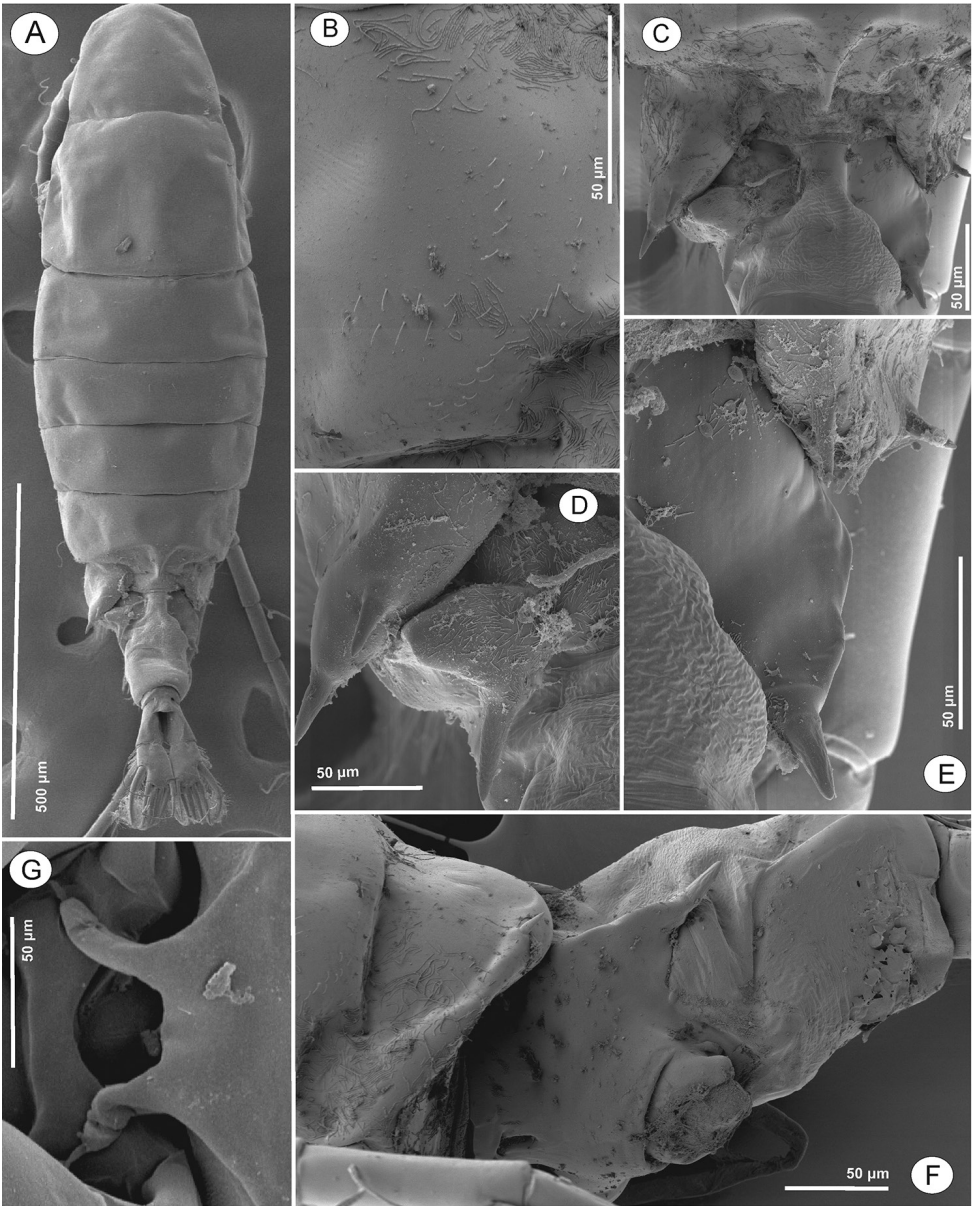
Phyllodiaptomus (P.) roietensis sp. nov., female: **A** habitus, dorsal view **B**Pdg 4, lateral view **C**Pdg 5 and genital double-somite, dorsal view **D**Pdg 5 and genital double-somite spines, lateral view from left side **E**Pdg 5 and genital double-somite spines, lateral view from right side **F**Pdg 5 and genital double-somite, lateral view **G** rostral spines.

**Figure 4.**
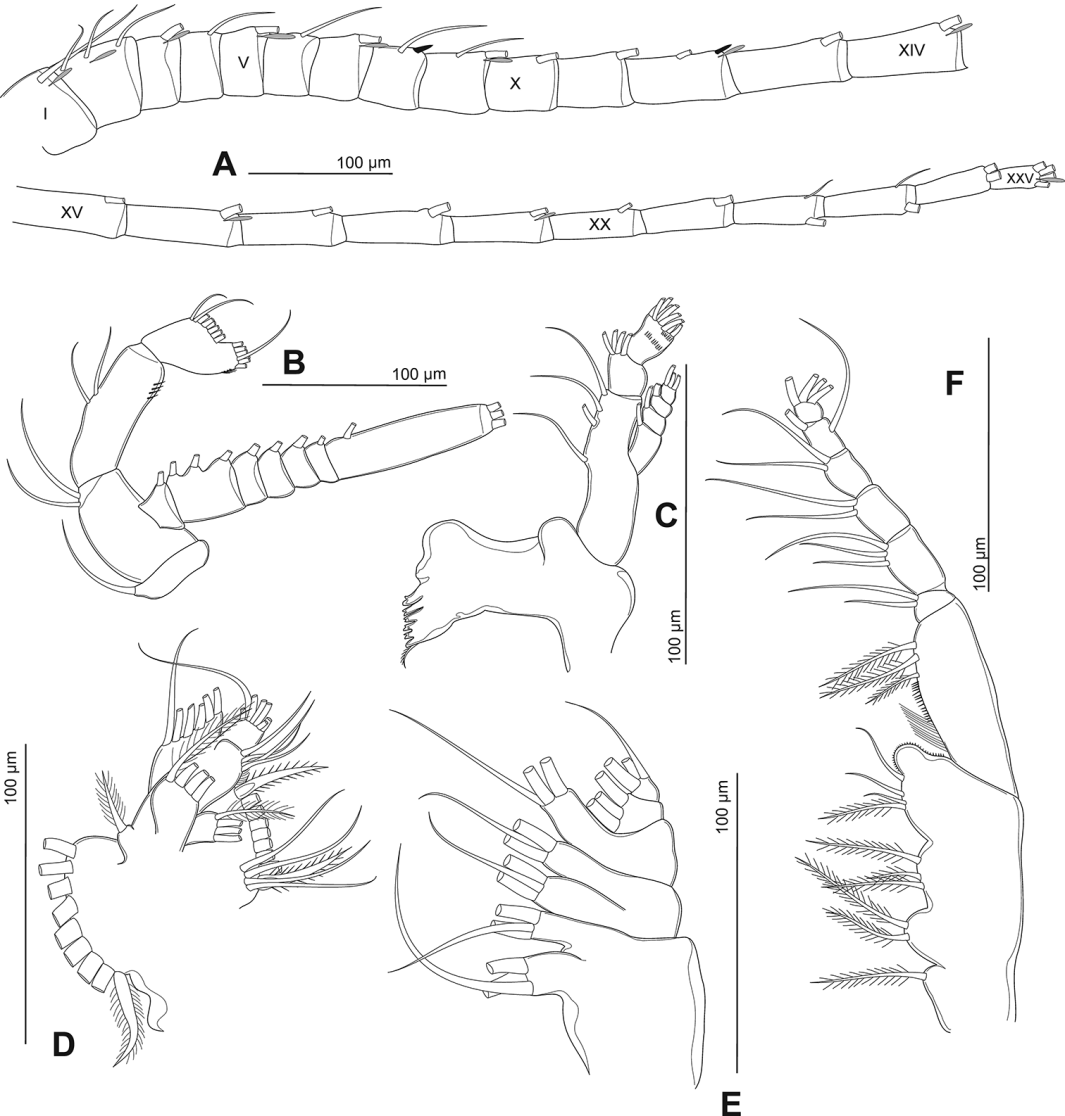
Phyllodiaptomus (P.) roietensis sp. nov., female: **A** antennule **B** antenna **C** mandible **D** maxillule **E** maxilla **F** maxilliped.

**Figure 5.**
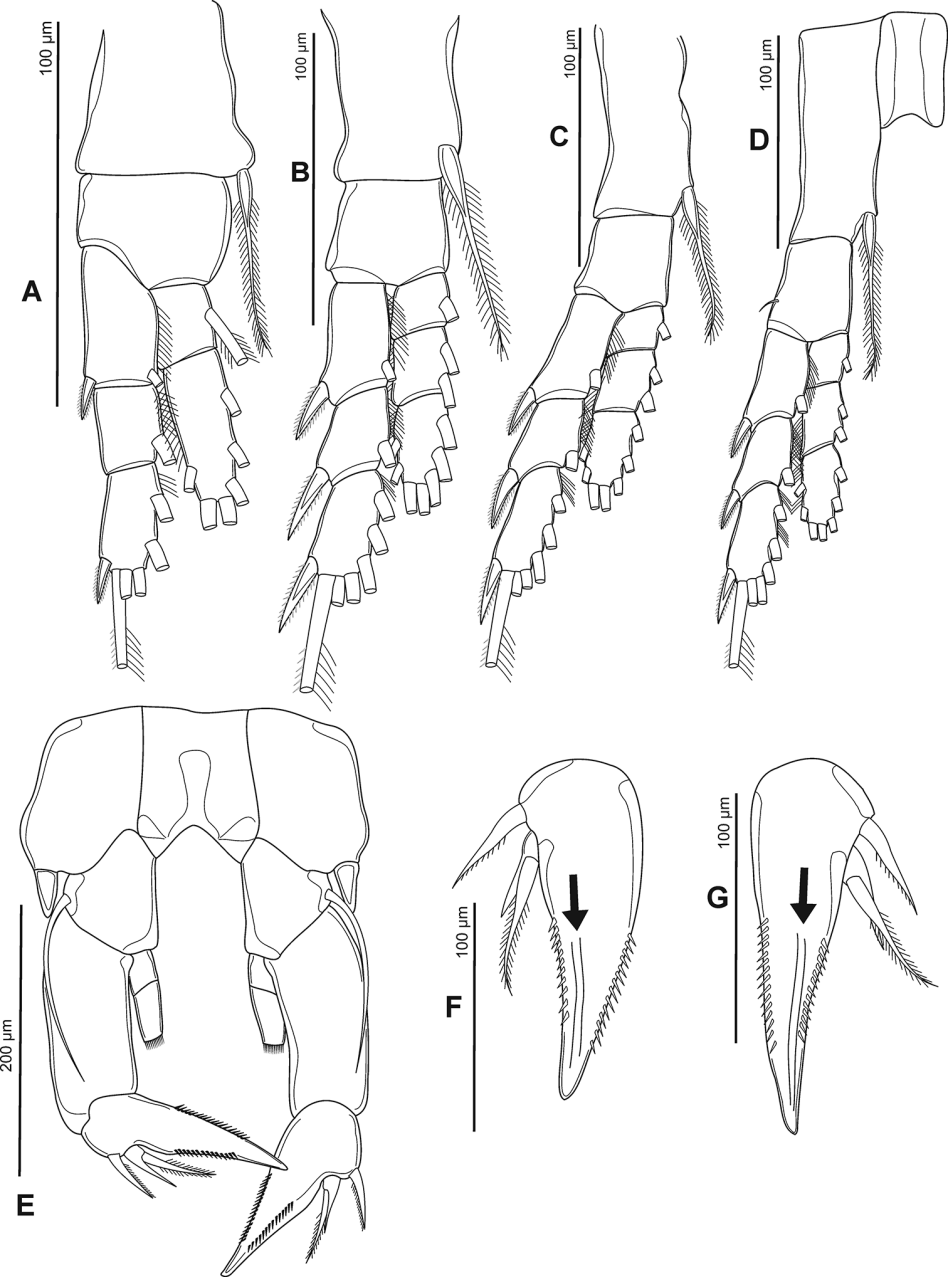
Phyllodiaptomus (P.) roietensis sp. nov., female: **A** P1 **B** P2 **C** P3 **D** P4 **E** P5 **F, G** right and left P5 Exp-2 (black arrows indicate longtitudinal ridges) **A–E** posterior view **F, G** anterior view.

**Figure 6.**
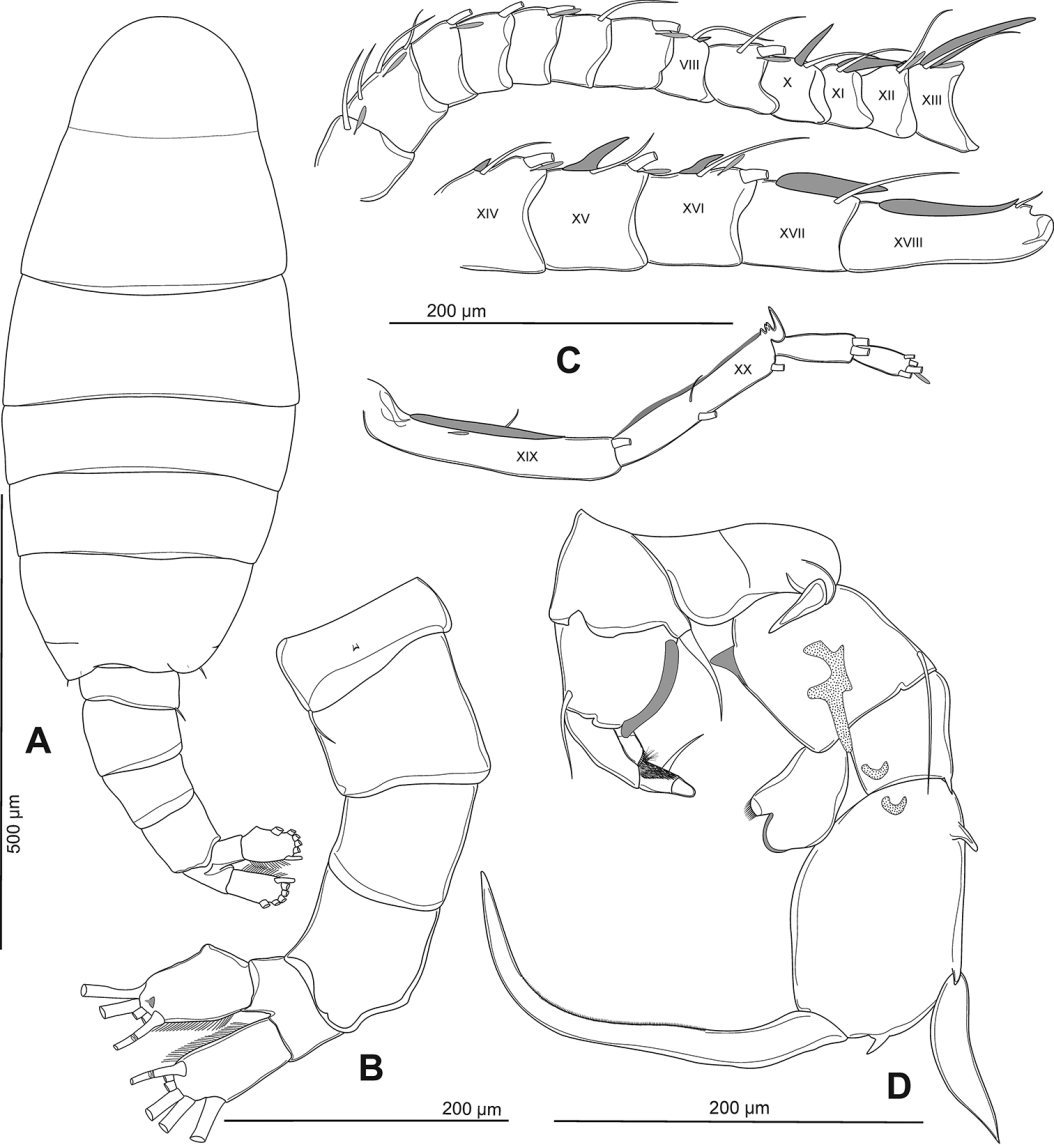
Phyllodiaptomus (P.) roietensis sp. nov., male: **A** habitus, dorsal view **B** urosome, ventral view **C** right antennule, with grey objects indicating antennular spines **D** P5, with grey and dotted objects indicating hyaline lamella and chitinous prominences respectively, posterior view.

**Figure 7.**
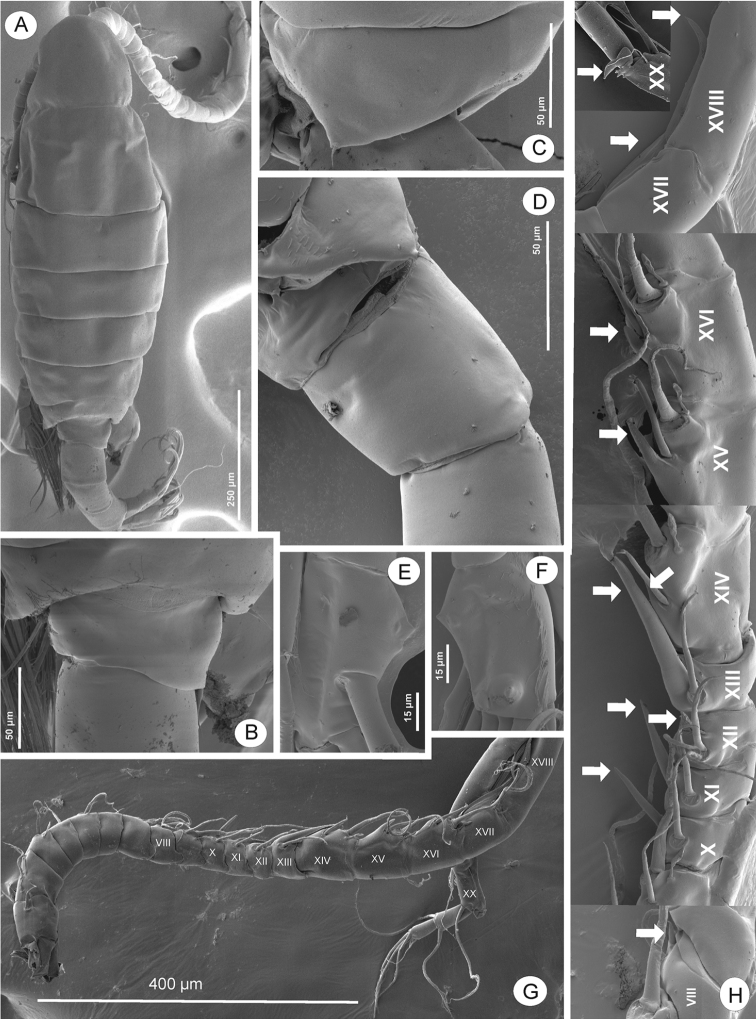
Phyllodiaptomus (P.) roietensis sp. nov., male: **A** habitus, dorsal view **B**Pdg 5 and genital somite, dorsal view **C**Pdg 5 lateral wing, left view **D**Pdg 5, genital somite and urosomites 2 and 3, ventrolateral view **E, F** right caudal ramus in dorsal (**E**) and ventral (**F**) views **G, H** right antennule (white arrows indicate spines on segments 8, 10–18, 20.

**Figure 8.**
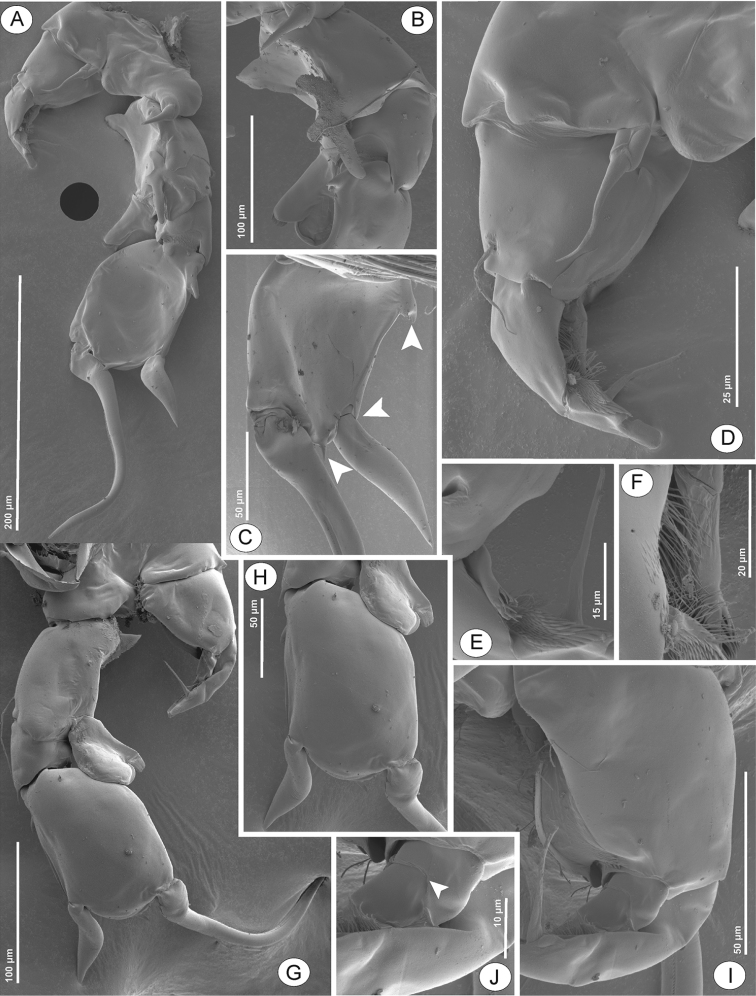
Phyllodiaptomus (P.) roietensis sp. nov., SEM photographs, male: **A** P5 in posterior view **B** right P5 basis, Exp-1 and Enp, posterior view **C** right P5 Exp-2 in posterior view (white arrows indicate accessory spines) **D** left lobe of P5, posterior view **E** left P5 Exp-2 and Enp, posterior view **F** left P5 Enp, posterior view **G** P5, anterior view **H** right P5 Exp-1–2 and Enp, anterior view **I** left P5 basis, Exp and Enp, anterior view **J** left P5 Enp (white arrow indicates Enp segmented point), anterior view.

Figs 2 , 3 , 4 , 5 , 6 , 7 , 8

###### Type locality.

A pool in the rice field at Ban Nakae, Khilek Subdistrict, Pathum Rat District, Roi Et Province, northeastern Thailand; pH of water 8.6, water conductivity 126 µS cm^-1^.

###### Type material.

***Holotype***: one adult male completely dissected (NHMUK 2019.7, one slide), Ban Nakae (15°37'37"N, 103°28'06"E), Khilek Subdistrict, Pathum Rat District, Roi Et Province, northeastern Thailand; collected on 12 June 1999 by L. Sanoamuang. ***Allotype***: one adult female completely dissected (NHMUK 2019.8, one slide); same data as for holotype. ***Paratypes***: two adult females and three adult males undissected preserved in 70% ethanol (NHMUK 2019.9–13), one adult female completely dissected (KKU-COP-2019-S-01); one adult female with eggs and three adult males undissected preserved in 70% ethanol (KKU-COP-2019-T-01); same data as for holotype.

###### Other localities.

(1) a temporary pond, Ban Non Lakki (15°10'55"N, 102°23'46"E), Than Lalot Subdistrict, Phimai District, Nakhon Ratchasima Province, northeastern Thailand; collected on 17 October 2017 by N. Plangklang; (2) a roadside canal, Tropeang Chouk village (no geographical co-ordinates), Baray District, Kampong Thom Province, central Cambodia; collected on 14 June 2007 by R. Chaicharoen; (3) a temporary pond, Kropeu village (no geographical co-ordinates), Steung Sen District, Kampong Thom Province, central Cambodia; collected on 14 June 2007 by R. Chaicharoen.

###### Description of adult female.

Total body length measured from anterior margin of rostrum to posterior margin of caudal rami, 0.9–1.3 mm. Rostrum (Fig. 3G) with bifid process in distal margin, pointed backward; each with short spine at tip. Prosome length: urosome plus caudal rami ratio about 2.6:1, ratio of width to length of prosome = 1:2.4, urosomites 1–3 = 1.3:3.0:1.1, caudal ramus = 1:1.5. Prosome (Figs 2A, 3A) ovoid, cephalosome with transversal groove in anterior part of somite length; Pdg 4 and 5 fused, partly separated laterally, with few tiny hair-like spinules scattered laterally (Fig. 3B, C). Pdg 5 (Figs 2C, E, 3C–F) with asymmetrical postero-lateral wings; right one rounded; left one longer and triangular; each wing with dorsal and posterior spines (former spine slightly larger than later one). Urosome (Figs 2A, 3A) with asymmetrical genital double-somite. Genital double-somite (Figs 2C–E, 3C–F) longer than urosomite 2, anal somite and caudal ramus combined. Left side with obviously laterally dilated proximal part of genital-double segment; dilatation dorsally with large and blunt spine distally, tip of spine oriented medially. Right side with slightly dilated proximal part of genital double-somite; elongated into triangular outgrowth with blunt spine at tip; spine orientated ventro-laterally. A pair of gonopores located beneath genital operculum, at about one-half length of genital double-segment. Adult female bears one egg sac with 20–25 eggs (Fig. 2A). Urosomite 2 symmetrical, very short. Anal somite (Figs 2C, D, 3A) as long as wide; anal operculum small, free margin convex. Caudal rami (Fig. 2A, D) symmetrical, with row of setules along inner and outer margins. Ramus with six setae (seta II–VII), subequal in length, all plumose but dorsal (VII); dorsal seta articulated, longest.

Antennule (Fig. 4A) symmetrical, 25-segmented, reaching beyond the end of caudal setae. Setal formula (Roman numerals in parentheses refer to segment number): 1+ae (I), 3+ae (II), 1+ae (III), 1(IV), 1+ae (V), 1 (VI), 1+ae (VII), 1+sp (VIII), 2+ae (IX), 1 (X), 1 (XI), 1+ae+sp (XII), 1 (XIII), 1+ae (XIV), 1 (XV), 1+ae (XVI), 1 (XVII), 1 (XVIII), 1+ae (XIX), 1 (XX), 1 (XXI), 2 (XXII), 2 (XXIII), 2 (XXIV), 4+ae (XXV).

Antenna (Fig. 4B) biramous. Coxa and basis with one and two inner setae on distal corner, respectively. Exp-1–7 with 1, 3, 1, 1, 1, 1, and 1 inner seta, respectively; Exp-7 with three additional apical setae. Enp-1 with two inner median setae. Enp-2 with eight inner and seven apical setae.

Mandible (Fig. 4C) with six strongly chitinized teeth and one dorsal seta on gnathobase. Basis with four inner setae. Enp-1 with four inner distal setae; Enp-2 with nine apical setae plus tiny spinules along posterior surface. Exp-1–4 with 1, 1, 1, 3 setae, respectively.

Maxillule (Fig. 4D) with 13 setae on praecoxal arthrite. Coxal endite and coxal epipodite with four and nine setae, respectively. Proximal and distal endites each with four setae; basal exite with a single seta. Enp with seven apical setae. Exp with six setae.

Maxilla (Fig. 4E) with two praecoxal and two coxal endites; each with three apical setae. Allobasis with three setae. Enp-1 and 2 with three setae each.

Maxilliped (Fig. 4F) with four endites on syncoxa, with 1, 2, 3, 4 apical setae respectively. Basis with three setae along median margin. Enp-1–6 with 2, 3, 2, 2, 2, 4 setae, respectively.

P1–P4 (Fig. 5A–D) with round and bare intercoxal sclerite. Coxa with inner seta. P1 basis with reduced outer seta. Exp longer than Enp, Exp and Enp three-segmented except P1 Enp bi-segmented. Armature formula of P1–P4 as follows (Arabic and Roman numerals indicate number of setae and spines, respectively; outer-inner or outer-apical-inner indicate seta/spine):

**Table:** 

	Coxa	Basis	Exp			Enp		
			1	2	3	1	2	3
P1	0-1	0-0	I-1	0-1	I-3-2	0-1	1-2-3	–
P2	0-1	0-0	I-1	I-1	I-3-3	0-1	0-2	2-2-3
P3	0-1	0-0	I-1	I-1	I-3-3	0-1	0-2	2-2-3
P4	0-1	1-0	I-1	I-1	I-3-3	0-1	0-2	2-2-3

P5 (Fig. 5E) asymmetrical. Coxa with blunt, stout spine on distal outer margin. Basis with thin, bare seta on distal outer margin, reaching beyond 3/4 of Exp-1. Exp-1 sub-rectangular, more than twice as long as wide, longer than Enp. Exp-2 triangular, right side stout and shorter than left one; with row of strong spinules along margins and proximolateral spine at basal Exp-3; with two longitudinal grooves on anterior view (Fig. 5F, G). Exp-3 represented by small distal prominence produced into short distolateral spine and longer medial spine. Enp subconical, Enp-1 slightly rectangular. Enp-2 tipped with circular row of spinules.

###### Description of adult male.

Body length (Figs 6, 7A) without caudal setae, 0.8–1.1 mm (mean = 1.0 mm, n = 5), smaller than female. Prosome length: urosome plus caudal rami ratio about 2.1:1, ratio of width to length of prosome = 1:2.1, urosomites 1–5 = 2.3:1.0:1.0:1.3:1.0, caudal ramus = 1:1.9. Prosome similar to that of female except lateral wings on Pdg 5. Lateral wings (Figs 6A, 7B–D) asymmetrical, round on right and more triangular on left side; posterior spine on right wing larger compared to left side. Urosome (Figs 6A, B, 7A) 5-segmented, asymmetrical, curved downward to right side. Genital somite (Figs 6, 7B) dilated postero-laterally on right side, with spine at distal outer corner; longer than that on Pdg 5 wings. Genital aperture located on mid-ventral region. Urosomites 2–3 (Figs 6B, 7D) without ornamentation. Urosomite 4 (Fig. 6A, B) with irregularly dilated posterior margin. Anal somite (Fig. 6A, B) asymmetrical and twisted to right side. Caudal rami (Figs 6A, B, 7E, F) asymmetrical, right ramus with two triangular prominences: one proximolateral and one distoventral; setation similar to female.

Antennule (Figs 6C, 7G, H) asymmetrical, with geniculated right side. Right antennule 22-segmented, with setal formula as 1+ae (I), 3+ae (II), 1+ae (III), 1 (IV), 1+ae (V), 1 (VI), 1+ae (VII), 1+sp (VIII), 2+ae (IX), 1+sp (X), 1+sp (XI), 1+ae+sp (XII), 1+ae+sp (XIII), 2+ae+sp (XIV), 2+ae+sp (XV), 2+ae+sp (XVI), 1+sp (XVII), 1+sp (XVIII), 2+ae+sp (XIX), 3+sp (XX), 2 (XXI), 4+ae (XXII); geniculated between segments 18 and 19; segment 20 (antepenultimate) with serrated process distally (3–4 teeth), and with longitudinal hyaline membrane along outer margin.

Left antennule, antenna, mouthparts, and P1–P4 as in female.

P5 (Figs 6D, 8A, G) intercoxal sclerite with rounded lobe on free margin. Right P5: coxa with acute, stout spine on posterior lobe. Basis (Fig. 8B, G) with large proximomedial triangular lamella at one-fourth length of inner margin; with large three-lobed chitinous medial prominence on posterior surface; distal outer margin with long, thin seta, slightly extending beyond Exp-1. Enp (Fig. 8B, H, G) with bi-lobed distal margin, tipped with spinules and hyaline lamella on inner and outer lobes, respectively; reaching downward to approximately one-third of Exp-2. Exp-1 (Fig. 8A, B, H) with semi-circular knob on distomedial margin; distolateral margin with small acute process. Exp-2 (Fig. 8C, H) elliptic, with three accessory lateral spines, one proximal, middle, and distal on lateral margin. Principal lateral spine articulated, located at two-third length of Exp-2, flat, thick, digitiform, with sharp tip; long, with approximately half of segment bearing it; slightly twisted in posterolateral direction. End claw (Figs 6D, 8A) medially sickle-shaped, slender towards tip, more than 1.5 times as long as Exp-2; medial margin serrated with row of tiny spinules.

Left P5 (Figs 6D, 8D): coxa with moderate strong seta inserted on posterior lobe at distal inner corner, slightly shorter than distal margin of basis. Basis with flap of longitudinal hyaline lamella at medial margin; with long, thin seta at posterolateral margin, extending to approximately half of Exp-1. Exp-1 (Fig. 8F) tapering towards posterior margin, medial margin concave, with field of setules and tiny spinules. Exp-2 smaller than Exp-1, conical; with large seta at mid-length of medial margin, as long as Exp-2 and apical process combined; with few setules proximally and widespread with spinules distally along inner margin, thickness of spinules increased from proximal to distal; apical process stout, bare, and blunt-tip. Enp (Fig. 8F, J) bi-segmented, longer than Exp-1, Enp-2 tipped with row of spinules distally.

###### Differential diagnosis.

Phyllodiaptomus (P.) roietensis sp. nov. with the male P5 Exp-2 displays an affinity to the subgenus Phyllodiaptomus sensu Dumont et al. (1996): the lateral side of the right Exp-2, medially concave in posterior view, principal lateral spine inserted on distal to mid-outer margin and three accessary spines arranged from proximal, middle, and distal, respectively; the left Exp-2 with patch of strong spinules along medial margin.

The male of the new species has serrated outgrowth on the antepenultimate segment of the right antennule. The right caudal ramus with small chitinous spine near distal margin on ventral side and triangular prominence along proximal one-third length of outer margin. The P5 intercoxal sclerite produced, with convex distal margin. The right P5 with (1) short, strong spine on posterior lobe of coxa, (2) triangular hyaline lamella on proximal inner margin and large chitinous outgrowth on posterior surface of basis, (3) acute distal outer corner of Exp-1 (4) Exp-2 oval and concave, with strong, flat, curved principal spine and three accessary spines, and (5) bi-lobed Enp. The left P5 with long and narrow hyaline lamella along inner margin, Exp-2 with patch of strong spinules along medial margin, and bi-segmented Enp.

Female with asymmetrical Pdg 5 wings, left wing more elongated in posterio-lateral direction; posterior and dorsal spines short and strong. Genital double-somite with posterolateral directed process on right side. One pair of genital spines on lateral side slightly symmetrical and strong. P5 Exp-2 with conveyor canal on anterior surface. P5 with bi-segmented Enp.

###### Etymology.

The specific name *roietensis* is taken after the type locality, Roi Et Province. The name with the Latin suffix “-*ensis*” is the adjective for a location.

###### Distribution.

Known only from four temporary water bodies from Roi Et and Nakhon Ratchasima provinces, Thailand and Kampong Thom Province, Cambodia (Fig. 1). Presence of specimens was recorded in early monsoon period. The new species is rare, as it was found in 0.4% of all the localities sampled in Cambodia. The new species was found together with six diaptomids including *Dentodiaptomus javanus* (Grochmalicki, 1915), *Eodiaptomus sanoamuangae* Ranga Reddy & Dumont, 1998, *Mongolodiaptomus calcarus* (Shen & Tai, 1965), *M. malaindosinensis* (Lai & Fernando, 1978), *Neodiaptomus laii* Kiefer, 1974, and Phyllodiaptomus (Phyllodiaptomus) christineae Dumont, Ranga Reddy & Sanoamuang, 1996.

## Discussion

To date, the genus *Phyllodiaptomus* has been recorded in Asia, including south China, Turkey, Israel, Uzbekistan, Iran, Iraq, India, Sri Lanka, Nepal, Indonesia, Thailand, Laos, Philippines and Cambodia (Dumont and Ranga Reddy 1993; Ranga Reddy 1994; Dumont et al. 1996; Ranga Reddy et al. 1998; Sanoamuang 1999; Sanoamuang and Yindee 2001; Sanoamuang and Teeramaethee 2006; Khalaf 2008; Alekseev et al. 2013, 2016; Marrone et al. 2014; Bekleyen et al. 2017; Guinto et al. 2018; Sanoamuang and Watiroyram 2018). Most species are considered endemic to specific countries. Three species (P. (C.) annae, P. (C.) wellekensae, and P. (C.) sasikumari) are endemic to India; two species (P. (P.) thailandicus and P. (P.) surinensis) are endemic to Thailand; P. (P.) tunguidus, P. (P.) irakiensis, and P. (P.) longipes are endemic to China, Iraq, and Indonesia, respectively. Only P. (P.) blanci is widely distributed, extending across many countries. Five species have been recorded in Thailand, namely P. (C.) praedictus, P. (P.) christineae, P. (P.) thailandicus, P. (P.) surinensis, and P. (P.) roietensis sp. nov. (Sanoamuang 2002; this study). Among the Thai sister species, P. (P.) surinensis and P. (P.) roietensis sp. nov. are rare. In 3,000 samples collected within Thailand, each has been recorded in only two localities in the northeast. This is in contrast to another endemic Thai species, P. (P.) thailandicus, which is widely distributed in both temporary and permanent water bodies in the east and south of Thailand (Sanoamuang 2002).

The right antennule is mainly used as a clasping organ in all males of the family Diaptomidae, and it normally bears spines or spinous processes on segments 8, 10–16, and 20 (Kulkarni et al. 2018). However, P. (P.) roietensis sp. nov., *Mongolodiaptomus loeiensis* Watiroyram & Sanoamuang, 2017, and *Mongolodiaptomus mekongensis* Sanoamuang & Watiroyram, 2018 differ from P. (P.) surinensis and other diaptomids by having additional spines on segments 17–19 (see Figs 5C, 6H; Watiroyram and Sanoamuang 2017: fig. 4F; Sanoamuang and Watiroyram 2018: fig. 6D). The males of these species may manage to mate more easily with females using the unique ornamentation of antennule and caudal ramus. In females, Pdg 5 wings and genital double-somites are probably important for species recognition and mating behavior of their males (Ohtsuka and Huys 2001; Ali et al. 2014). Although the male morphological features of the two parapatric *Phyllodiaptomus* are different, they are able to differentiate their conspecific females during mating, as the females of the new species can be distinguished from its congeners by the presence of posterolateral process on both sides of genital double-somite, which are absent in other congeners except P. (P.) thailandicus. However, the characteristic that differentiates the new diaptomid from P. (P.) thailandicus is the presence of a single process on each side of the genital double-somite; P. (P.) thailandicus has two processes only on the right side (Figs 2C–E, 3C–F; Sanoamuang and Teeramathee 2006: figs 1, 24). In contrast to their males, the new species and P. (P.) surinensis have unique females which can be easily differentiated. The female P5 Exp-2 of the new species is obviously asymmetrical compared with that of P. (P.) surinensis which has a slightly asymmetrical P5 Exp-2. Dumont and Ranga Reddy (1993) observed that the conveyor canal on the P5 Exp-2 in females is species-specific and unique to the genus *Phyllodiaptomus*: the new species has two longitudinal ridges on the anterior surface versus multi-longitudinal ridges in P. (P.) surinensis (Fig. 5F, G; Sanoamuang and Yindee 2001: fig. 39). The clasping site on the genital double-somite of the new species is wider than those in P. (P.) surinensis. The new species has substantial left genital double-somite proximal bulging versus only slight asymmetry in P. (P.) surinensis. The genital double-somite of P. (P.) surinensis has a bi-lobed hyaline outgrowth ventrally, which is absent in the new species. The genital spines in the female of the new species are oriented to a posterolateral direction in dorsal view, whereas they are pointed to the lateral direction in P. (P.) surinensis. The new species has tiny spinules on Pdg 4–5 laterally; these are present dorsally in P. (P.) surinensis (Fig. 3B; Sanoamuang and Yindee 2001: fig. 2).

The male of P. (P.) roietensis sp. nov. has a number of morphological differences from other members of the *blanci*-species group as follows:

a) Antepenultimate segment with a serrated process versus smooth in P. (P.) longipes.

b) Urosomite(s) with a long hair or hair-like setae versus bare in P. (P.) thailandicus, P. (P.) christineae, and P. (P.) blanci.

c) Right caudal ramus with ventral prominences as in P. (P.) surinensis and P. (P.) tunguidus. However, a ventral prominence is also present on the left ramus of P. (P.) tunguidus (but is absent in the left ramus of the new species) and there are only two prominences in the new species but five in P. (P.) surinensis.

d) Intercoxal sclerite is modified distally into single lobe versus two lobes in P. (P.) irakiensis and P. (P.) thailandicus. The new species has a round or semi-circular distal margin versus triangular in P. (P.) blanci, P. (P.) christineae, P. (P.) longipes, and P. (P.) tunguidus.

e) Right P5 coxal spine is strong and acute versus rectangular in P. (P.) thailandicus and slender in P. (P.) christineae.

f) Right P5 basis with a three-lobed chitinous prominence on posterior surface versus bare in P. (P.) irakiensis and P. (P.) blanci. In addition, three species, P. (P.) longipes, P. (P.) christineae, and P. (P.) tunguidus, have a longitudinal ridge on the posterior surface, which is absent in the new species (the first one has two minute prominences on the ridge). The right P5 basis has a triangular hyaline lamella at inner margin versus elongated in P. (P.) christineae, P. (P.) longipes, and P. (P.) tunguidus, and round in P. (P.) blanci. The left P5 basis has inner lamella versus bare in P. (P.) irakiensis and P. (P.) longipes, digitiform in P. (P.) tunguidus, and small in P. (P.) blanci. The new species lacks any ornamentation on the anterior surface but P. (P.) surinensis has two minute lateral spines (see Sanoamuang and Yindee 2001: fig. 54).

g) Right P5 Exp-2 with three accessary lateral spines versus bare in P. (P.) tunguidus and P. (P.) blanci, and one in P. (P.) irakiensis, P. (P.) christineae, and P. (P.) longipes.

h) Right P5 Enp with a bi-lobed shape versus conical in the rest of the species except P. (P.) surinensis.

i) Left P5 with bi-segmented Enp versus one-segmented in P. (P.) thailandicus, P. (P.) surinensis, P. (P.) blanci, P. (P.) christineae, and P. (P.) longipes.

With regard to the comparative morphology above, the male of the new species is most similar to those of P. (P.) surinensis. However, there are three major differences among the males, i.e. the right caudal ramus, left P5 basis, and left P5 Enp as described above. The fine detail on its inner hyaline lamella on the right P5 basis is also different: triangular in the new species versus oval bi-lobed in P. (P.) surinensis.

Ranga Reddy (1994) provided the first key to species and included six species of *Phyllodiaptomus* (P. (P.) tunguidus, P. (P.) blanci, P. (P.) longipes, P. (C.) annae, P. (C.) wellekensae, and P. (C.) sasikumari); he also gave morphological descriptions of these six species. In this study, the key is updated as follows:

### Keys to worldwide species of *Phyllodiaptomus* Kiefer, 1936

**Males**:

**Table:** 

1	Left P5 Exp-2 with a serrate hyaline fan on inner margin	**2 (subgenus Ctenodiaptomus)**
–	Left P5 Exp-2 with a field of spinules on inner margin	**5 (subgenus Phyllodiaptomus)**
2	Inner margin of P5 intercoxal sclerite with conical lobe, blunt tip	**P. (C.) sasikumari**
–	Inner margin of P5 intercoxal sclerite with triangular lobe, acute tip	**3**
3	Right P5 Exp-1 without acute process on distal outer corner	**P. (C.) wellekensae**
–	Right P5 Exp-1 with acute process on distal outer corner	**4**
4	Right P5 Exp-2 without hyaline lobe on distal outer corner	**P. (C.) praedictus**
–	Right P5 Exp-2 with hyaline lobe on distal outer corner	**P. (C.) annae**
5	Antepenultimate segment with smooth process	**P. (P.) longipes**
–	Antepenultimate segment with serrated process	**6**
6	Urosomite 2–3 or only 2 with hair or hair-like setae	**7**
–	Urosomite 2–3 without hair or hair-like setae	**9**
7	Inner margin of P5 intercoxal sclerite with two lobes	**P. (P.) thailandicus**
–	Inner margin of P5 intercoxal sclerite with triangular lobe	**8**
8	Right P5 Exp-2 with slender principal spine	**P. (P.) christineae**
–	Right P5 Exp-2 with thick principal spine	**P. (P.) blanci**
9	Inner margin of P5 intercoxal sclerite with two lobes	**P. (P.) irakiensis**
–	Inner margin of P5 intercoxal sclerite with single lobe	**10**
10	Right P5 Exp-1 without acute process on distal outer corner	**P. (P.) tunguidus**
–	Right P5 Exp-1 with acute process on distal outer corner	**11**
11	Right P5 basis with one-lobed hyaline lamella on inner margin	**P. (P.) roietensis sp. nov.**
–	Right P5 basis with two-lobed hyaline lamella on inner margin	**P. (P.) surinensis**

**Females**:

**Table:** 

1	Genital double-somite with postero-laterally oriented outgrowth	**2**
–	Genital double-somite without postero-laterally oriented outgrowth	**3**
2	Genital double-somite with two postero-laterally oriented outgrowths on right side	**P. (P.) thailandicus**
–	Genital double-somite with single postero-laterally oriented outgrowth on right side	**P. (P.) roietensis sp. nov.**
3	P5 Enp one-segmented	**P. (P.) longipes**
–	P5 Enp two-segmented	**4**
4	Pdg 5 left wing bi-lobed	**5**
–	Pdg 5 left wing round or triangular	**6**
5	Pdg 5 wings symmetrical	**P. (P.) tunguidus**
–	Pdg 5 wings asymmetrical	**P. (P.) irakiensis**
6	Pdg 5 right wing round or triangular	**7**
–	Pdg 5 right wing bi-lobed	**8**
7	Genital double-somite with ventral hyaline outgrowth	**P. (P.) surinensis**
–	Genital double-somite without ventral hyaline outgrowth	**P. (P.) christineae**
8	Genital double-somite dilated at the proximal left side	**9**
–	Genital double-somite non-dilated at the proximal left side	**10**
9	Genital double-somite dilated at the middle of right side	**P. (C.) praedictus**
–	Genital double-somite non-dilated at the middle of right side	**P. (C.) wellekensae**
10	Genital double-somite dilated at the middle of right side	**P. (P.) blanci**
–	Genital double-somite non-dilated at the middle of right side	**11**
11	P5 basis with short lateral seta, not reaching over Exp-1	**P. (C.) annae**
–	P5 basis with long lateral seta, reaching over Exp-1	**P. (C.) sasikumari**

## Supplementary Material

XML Treatment for Phyllodiaptomus (P.) roietensis
